# Porcelain Teeth

**Published:** 1842-03

**Authors:** 


*Porcelain Teeth.*—The manufacture of these teeth have, within the last few years, been brought to very great perfection. Although invented and manufactured for a long time in France previously to their introduction into America, those which are now obtained from that country do not bear any thing like so close a resemblance, either in shape or colour, to the natural organs, as those that are manufactured in this. We believe that the porcelain teeth which are manufactured in the United States, and especially in Philadelphia and New York, are superior, in almost every respect, to those that are manufactured in any other part of the world. This is saying a great deal, but we believe we speak understandingly upon the subject. We must however, do Mr. Ash, of London, the justice to say, and at the same time we beg leave to return him our thanks for the very beautiful specimens of teeth which he did us the courtesy to send us a few months since, that the teeth which he manufactures so far as their shape and resemblance in all other respects to the natural teeth are concerned, are unsurpassed by any which we have ever seen. But they have one fault, and that is, they do not stand the degree of heat necessary to attach them to gold plate with solder sufficiently pure to resist the action of the fluids of the mouth. With American practitioners this is an insuperable objection.

But notwithstanding the perfection to which the porcelain teeth have been brought, important improvements in them, are almost every day being made. A few months since we saw a very important improvement in the manner of securing the platina rivets to plate teeth, by Dr. Harrington of Philadelphia. It consisted in forming heads upon them previously to introducing them into the porcelain paste, and by which their liability to come out, is, we should suppose, effectually prevented.

While on a visit to New York, about a year and a half ago, we were shown some very beautiful teeth, by our friend Dr. L. Parmly, manufactured by Mr. Alcock, of that city. If they had any faults, we were unable to detect them.

The manufacture of these teeth, however, has been and is at present, carried on more extensively, we believe, by Mr. S. W. Stockton, of Philadelphia, than any other gentleman in this country. The teeth manufactured by him are known and used in almost every part of the world where dental surgery is practised, and to him the profession are very greatly indebted. To supply them with an article that should be lasting, and resemble as nearly as possible the human teeth, he has laboured assiduously and perseveringly for many years, and his efforts have certainly been crowned with a success flattering to himself and gratifying to the profession. Recently, he has made a very valuable and highly important improvement, consisting in the formation of an artificial gum to single teeth, so nearly resembling the natural, that, when placed in the mouth, none but a close and critical observer can perceive the difference. To the dental surgeon, and many requiring artificial teeth, this is a desideratum, for it often happens that the loss of the teeth is either accompanied, or followed by the destruction of the alveolar ridge, and to remedy which, the practice hitherto, when porcelain teeth were used, has been to form them in a block so as to fit as accurately as possible to the parts that it was to rest upon. This plan, however, for various reasons has been found objectionable. In the first place, block teeth, as they are usually called, are too clumsy to be worn with comfort. In the second, they are more liable to get broken, than single teeth mounted upon gold plate, and in the third and last place, they cannot be fitted as accurately to the inequalities of the parts upon which they are to rest as single teeth fitted to a properly adapted metalic plate.

We are aware that single teeth with gums have been manufactured for a long time in Paris, and we have a number of them at this time in our possession, but the imitation, both of the teeth and gums, is so poor that we have seldom used them. In these respects, the specimens which we have seen from Mr. Stockton's manufactory, are faultless, but that they are susceptible of improvement in some others, is very probable, and we hope he will not cease his efforts until he shall have perfected them in all

There are other gentlemen in Philadelphia and New York, who have the reputation of manufacturing very excellent porcelain teeth. We hope they may meet with encouragement commensurate with their merits.

We intend to notice hereafter, such improvements in this department of the art, as shall from time to time be made, and that we may be the better enabled to do this, we would be glad, if manufacturers on making any which they may conceive to be valuable, would communicate them to us. It will be to their interest to do so.—*Balt. Ed.*

